# SARS-CoV-2–specific memory B cells can persist in the elderly who have lost detectable neutralizing antibodies

**DOI:** 10.1172/JCI152042

**Published:** 2022-01-18

**Authors:** Anna Jeffery-Smith, Alice R. Burton, Sabela Lens, Chloe Rees-Spear, Jessica Davies, Monika Patel, Robin Gopal, Luke Muir, Felicity Aiano, Katie J. Doores, J. Yimmy Chow, Shamez N. Ladhani, Maria Zambon, Laura E. McCoy, Mala K. Maini

**Affiliations:** 1 Division of Infection and Immunity, Institute of Immunity and Transplantation, University College London (UCL), London, United Kingdom.; 2 Virus Reference Department, Public Health England (now called UK Health Security Agency [UKHSA]), London, United Kingdom.; 3 Blizard Institute, Queen Mary University of London, London, United Kingdom.; 4 Immunisation and Countermeasures Division, Public Health England (now called UKHSA), London, United Kingdom.; 5 Department of Infectious Diseases, School of Immunology and Microbial Sciences, King’s College London, London, United Kingdom.; 6 London Coronavirus Response Cell, Public Health England (now called UKHSA), London, United Kingdom.

**Keywords:** COVID-19, Immunology, Adaptive immunity, Immunoglobulins

## Abstract

Memory B cells (MBCs) can provide a recall response able to supplement waning antibodies (Abs) with an affinity-matured response better able to neutralize variant viruses. We studied a cohort of elderly care home residents and younger staff (median age of 87 years and 56 years, respectively), who had survived COVID-19 outbreaks with only mild or asymptomatic infection. The cohort was selected because of its high proportion of individuals who had lost neutralizing antibodies (nAbs), thus allowing us to specifically investigate the reserve immunity from SARS-CoV-2–specific MBCs in this setting. Class-switched spike and receptor-binding domain (RBD) tetramer–binding MBCs persisted 5 months after mild or asymptomatic SARS-CoV-2 infection, irrespective of age. The majority of spike- and RBD-specific MBCs had a classical phenotype, but we found that activated MBCs, indicating possible ongoing antigenic stimulation or inflammation, were expanded in the elderly group. Spike- and RBD-specific MBCs remained detectable in the majority of individuals who had lost nAbs, although at lower frequencies and with a reduced IgG/IgA isotype ratio. Functional spike-, S1 subunit of the spike protein– (S1-), and RBD-specific recall was also detectable by enzyme-linked immune absorbent spot (ELISPOT) assay in some individuals who had lost nAbs, but was significantly impaired in the elderly. Our findings demonstrate that a reserve of SARS-CoV-2–specific MBCs persists beyond the loss of nAbs but highlight the need for careful monitoring of functional defects in spike- and RBD-specific B cell immunity in the elderly.

## Graphical abstract

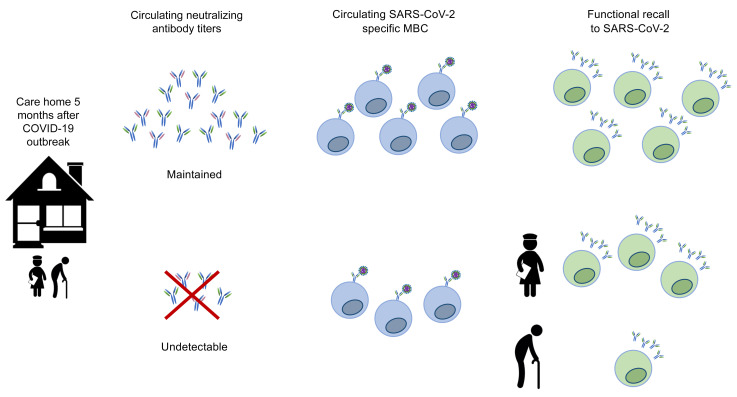

## Introduction

The human coronavirus SARS-CoV-2 has had a particularly devastating impact on the elderly, who are at much greater risk of morbidity and mortality (1, 2). Understanding the nature of a successful immune response in those who have avoided these outcomes and cleared SARS-CoV-2 after a mild infection, despite advanced age, is key to protecting this vulnerable group in the future. Whether older survivors of SARS-CoV-2 infection are able to mount robust and durable responses with the potential to provide long-term protection from reinfection, and from emerging viral variants, remains to be understood. Insights into the strengths and limitations of the immune response in those who have had a successful outcome of natural infection can inform the future optimization of vaccines. It is also crucial to understand the nature of the immune protection afforded to previously infected individuals while they await vaccination, especially with the ongoing delays in rollout and the lag in provision of vaccines to low- and middle-income countries.

Antibodies (Abs), in particular the neutralizing fraction, provide a vital frontline defense to achieve protective immunity against viruses. An initial waning of antibody titers is typically seen after resolution of an acute viral infection (3, 4). In the case of some viruses, long-lived plasma cells are then able to maintain Abs for decades (5–7). By contrast, in the months following infection with other viruses, including human coronaviruses like SARS-CoV-2, neutralizing Abs (nAbs) continue to wane and can drop below the threshold of detection in a proportion of individuals (3, 8–13). Even if Abs are maintained, they may fail to provide sufficient functional flexibility to cross-recognize viral variants (14–16). However, inadequate Ab titers or Abs that are unable to cross-recognize variants can be compensated by a second line of defense provided by antigen-specific memory B cells (MBCs) that are poised to react rapidly upon pathogen reencounter or vaccine boosting (17–19). Not only can MBCs provide a faster response upon reexposure to the virus, they are also able to diversify in the face of a mutating virus, resulting in a more potent, affinity-matured Ab response and enhanced resistance to viral mutations (9, 20).

In this study, we therefore sought to determine whether MBCs develop in elderly individuals following the resolution of SARS-CoV-2 infection and whether they can maintain functionality once the nAbs have waned. To address these questions, we studied elderly individuals with mild or asymptomatic SARS-CoV-2 infection who had recovered from the infection following outbreaks in 3 elder care homes in the United Kingdom. A substantial proportion of these individuals had lost detectable nAbs 5 months after infection. We compared MBCs between the elderly care home residents and younger staff members to assess the impact of aging. We identified MBCs specific for SARS-CoV-2 spike and receptor-binding domain (RBD) proteins that persisted when serum nAbs had waned below detectable limits. Their frequency, phenotype, isotype, and function were analyzed according to the individual’s age and/or nAb loss, to inform the assessment and boosting of durable immunity in the elderly.

## Results

### SARS-CoV2 spike- and RBD-specific MBCs can persist after loss of nAbs.

**Figure 1:**
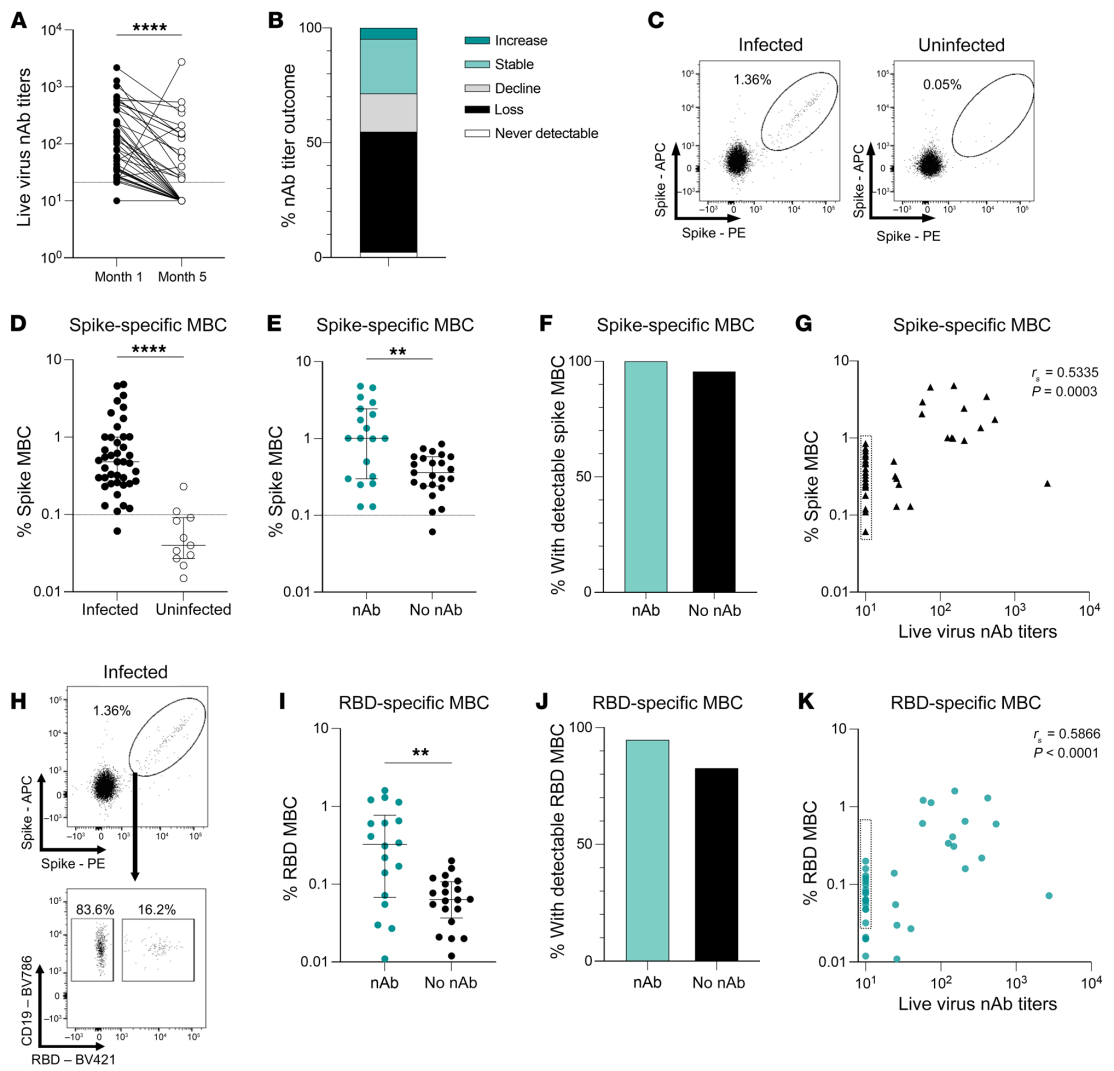
Spike- and RBD-specific MBCs persist 5 months after SARS-CoV-2 infection despite waning nAbs. (**A**) Paired live virus nAb titers 1 month and 5 months after infection (*n =* 42). (**B**) Proportion of infected individuals with a change in nAbs between months 1 and 5: increase = 4-fold or greater rise; static = less than a 4-fold increase/decrease; decline = 4-fold or higher decrease; loss = undetectable at 5 months; never detectable = undetectable at 1 month and 5 months (*n =* 42). (**C**) Representative FACS plots of dual staining of MBCs with SARS-CoV-2 spike tetramers for infected and uninfected individuals. (**D **and** E**) Frequency of spike-specific MBCs (**D**) in infected (*n =* 42) and uninfected (*n =* 11) individuals and in (**E**) infected individuals with nAbs (*n =* 19) or no nAbs (*n =* 13) at 5 months. Dashed lines indicate the threshold for spike-specific responses determined by pre-pandemic controls (see also Supplemental Figure 1B). (**F**) Proportion of infected individuals with detectable spike-specific MBCs with nAbs (*n =* 19) or no nAbs (*n =* 23) at month 5. (**G**) Correlation between the frequency of spike-specific MBCs and nAb titers (*n =* 42). (**H**) Representative FACS plots showing dual staining of MBCs with SARS-CoV-2 spike tetramers (top) and RBD tetramer staining of dual spike-specific MBCs (bottom) from an infected individual. (**I**) Frequency of RBD-specific MBCs in infected individuals with spike-specific responses stratified by nAbs (*n =* 18) and no nAbs (*n =* 20) at 5 months. (**J**) Proportion of infected individuals with RBD-specific MBCs with nAbs (*n =* 18) or no nAbs (*n =* 20) at 5 months. (**K**) Correlation between RBD-specific MBC frequency and live virus nAb titers (*n =* 38). (**A**) Wilcoxon matched-pairs test, *P ≤* 0.0001. (**D**,** E**, and** I**) Bars indicate the median and IQR; Mann-Whitney *U* test; (**D**) *P ≤* 0.0001, (**E**) *P =* 0.0039, (**I**) *P =* 0.003. (**F** and **J**) Fisher’s exact test; (**F**) *P* > 0.9999, (**J**) *P* = 0.6135. (**P < 0.005 and ****P < 0.0001.) (**G** and **K**) Dot-outlined boxes indicate individuals with discordant MBC and nAb responses. Significance was determined by Spearman’s rank correlation. Analysis of RBD-specific MBCs was done only for those with 20 or more cells in the spike-specific gate.

To study the role of MBCs, we obtained PBMCs from a subset of individuals (*n =* 42) from a large cohort who had survived COVID-19 with mild or asymptomatic infection after outbreaks in 3 elder care homes in April 2020 (see Methods, Supplemental Table 1, and refs. 21, 22). The care home cohort subset was selected in order to have a wide range of detectable titers of nAbs against live virus at the first sampling time point (month 1, May 2020, Figure 1), while all maintained detectable binding Abs by at least 1 assay (Supplemental Table 2; supplemental material available online with this article; https://doi.org/10.1172/JCI152042DS1). By the end of September 2020 (month 5), 29% of all participants sampled had stable (or in 2 cases increasing) nAbs against live virus. In contrast, 17% had declining titers, and 52% had lost detectable nAbs (Figure 1, A and B). One individual never had detectable nAb titers.

To compare MBC frequencies among individuals who had maintained or lost nAbs, we stained PBMCs with SARS-CoV-2 spike trimer tetramers, made by preincubating recombinant biotinylated trimeric spike protein with fluorescence-conjugated streptavidin (15). Dual staining with spike tetramers with 2 distinct fluorochromes was used to enhance the discrimination of true antigen-specific MBCs (Figure 1C), as described previously (23–25). We calculated the frequencies of antigen-specific responses within the memory fraction of B cells (CD19^+^CD20^+^ excluding IgD^+^, CD38^hi^, and CD21^+^CD27^–^ naive fractions; see gating strategy in Supplemental Figure 1A, as previously described in ref. 26). A threshold for background nonspecific staining was set at the mean ± 2 SD of staining seen in pre-pandemic healthy donor samples (Supplemental Figure 1B). Results were also compared with the control cohort derived from the same care homes (seronegative at both time points, Supplemental Table 1).

Spike-specific MBCs were detectable in 41 of the 42 tested individuals 5 months after infection, compared with 2 of 11 individuals of the care home control group who remained negative for binding Abs (Figure 1D). The frequency of spike-specific MBCs was reduced in those who had lost nAbs compared with those in whom they were still detectable (Figure 1E). Of note, however, most of those (96%) who had lost detectable nAbs still had some persistent spike-specific MBCs, a proportion comparable to that in the group that maintained nAb levels (Figure 1F). The frequency of spike-specific MBCs correlated significantly with the strength of the nAb response (nAb titer against live virus) at 5 months (Figure 1G); however, there was partial discordance due to detection of spike-specific MBCs in most individuals with no nAbs (dot-outlined box, Figure 1G).

Next, we analyzed the MBC response specifically directed against RBD, since this is the region within spike that many SARS-CoV-2–specific nAbs target (15, 27–29). RBD-specific MBCs were identified by gating on dual spike-tetramer staining cell populations that were also stained with a tetramer formed from recombinant biotinylated RBD protein preincubated with fluorescence-conjugated streptavidin (Figure 1H). RBD-specific responses were detectable in 38 of the 41 with a sufficient magnitude of spike-specific MBC responses (>20 dual spike^+^ cells) to allow analysis of the RBD-costained cells (Figure 1I). The frequency of RBD-specific MBCs was significantly reduced in the group of individuals who had lost nAbs compared with those with stable (or waning but still detectable) nAbs (Figure 1I). However, as noted with spike-specific MBCs, some RBD-specific MBCs remained detectable in most of the cohort, irrespective of whether they had lost nAbs (Figure 1J). Overall, the magnitude of RBD-specific MBCs correlated with nAb titers, although, again, there was some discordance due to the persistence of RBD-specific MBCs in those who had lost nAbs (dot-outlined box in Figure 1K). Importantly, both the RBD-positive and RBD-negative components of the spike-specific B cell response significantly correlated with nAb titers (Figure 1K and Supplemental Figure 1C). This highlights the importance of the RBD as the major target for nAbs, while also underscoring the contribution of Ab-targeting regions outside of the RBD (for example, the N-terminal domain [NTD] of the spike protein; refs. 15, 29–31) to the nAb response at the 5-month time point in this cohort.

These data therefore revealed the persistence of detectable, albeit reduced, MBCs specific for both spike and RBD proteins in most individuals whose nAb titers against live virus had fallen below the threshold of detection. Thus, loss of detectable nAbs 5 months after asymptomatic/mild infection is frequently compensated by the presence of a memory response primed to respond upon reexposure.

### Comparable persistence of spike- and RBD-specific MBCs in elderly care home residents and younger staff.

**Figure 2:**
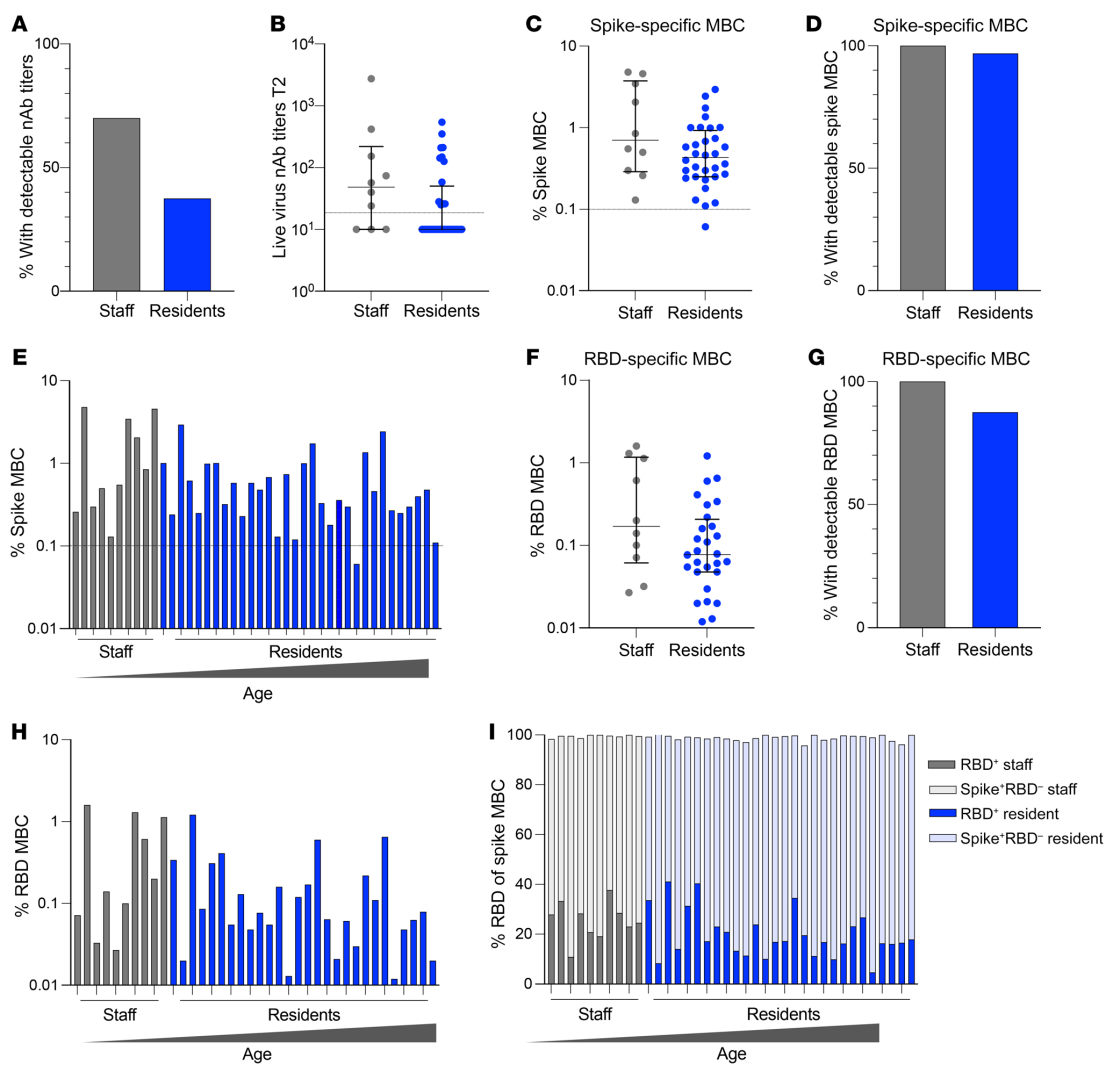
Comparable persistence of spike- and RBD-specific MBCs in elderly care home residents and younger staff. (**A**) Proportion of staff (*n =* 10) and residents (*n =* 32) with detectable nAbs at 1 month, who continued to have detectable nAbs at 5 months. (**B**) nAb titers at month 5 for all infected individuals stratified by staff (*n =* 10) and residents (*n =* 32). Dashed line indicates the assay threshold for detection; undetectable titers were assigned a value of 10. (**C**) Frequency of dual spike-specific MBCs in staff (*n =* 10) and residents (*n =* 32). (**D**) Proportion of infected individuals with detectable spike-specific MBCs stratified by staff (*n =* 10) and residents (*n =* 32). (**E**) Frequency of dual spike-specific MBCs for staff (gray) and residents (blue) ordered by age from youngest on the left to oldest on the right. (**F**) Frequency of RBD-specific MBCs in staff (*n =* 10) and residents (*n =* 28) with detectable spike-specific responses. (**G**) Proportion of infected individuals with detectable RBD-specific MBCs stratified by staff (*n =* 10) and residents (*n =* 32). (**H**) Frequency of RBD-specific MBCs for staff (gray) and residents (blue) ordered by age from youngest (left) to oldest (right). (**I**) Proportion of dual spike-specific cells with specificity for RBD (staff = dark gray; residents = dark blue) or the non-RBD region (staff = light gray; residents = light blue) in staff (*n =* 10) and residents (*n =* 28). (**A**,** D**, and **G**) Fisher’s exact test was used to determine statistical significance: (**A**) *P* > 0.9999, (**D**) *P* > 0.9999, and (**G**) *P =* 0.5569. (**B**, **C**, and** F**) Bars indicate the median and IQR. A Mann-Whitney *U* test was used to determine significance: (**B**) *P =* 0.4367, (**C**) *P =* 0.2552, and (**F**) *P =* 0.1068. (**C** and** E**) Dashed line indicates the threshold for spike-specific responses determined by pre-pandemic controls (Supplemental Figure 1B).

The elderly care home cohort was constructed to sample 2 comparator groups: elderly residents (median age, 87 years; range, 66–100 years) and a control group of younger staff (median age, 56 years; range 41–65 years). Five months after asymptomatic or mild infection, the proportion of elderly residents who had lost detectable nAbs was nonsignificantly lower than that of the care home staff members (Figure 2A), and those who maintained detectable nAbs had similar titers (Figure 2B). We postulated that there may, nevertheless, be a defect in the maintenance of spike- and RBD-specific MBCs in the elderly compared with the younger age group. However, spike-specific MBCs were maintained at similar frequencies and in comparable proportions in the elderly residents and younger staff (Figure 2, C and D). There were no clear trends toward a decrease in spike-specific MBCs with increasing age, even in residents in their nineties (Figure 2E). The percentage of global B cells (among live cells) was significantly lower in the elderly residents (in line with previous reports of B cell lymphopenia with aging; Supplemental Figure 2A) (32). However, residents had higher proportions of IgD^–^ MBCs than did staff members (Supplemental Figure 2B), such that spike-specific MBCs were still not significantly lower in residents than in staff when calculated as a percentage of all live cells acquired (Supplemental Figure 2C).

Similarly, RBD-specific MBCs were equally well maintained in the elderly residents and staff (Figure 2, F and G), with no decline in their frequencies (as a fraction of total MBCs) with increasing age (Figure 2H). RBD-specific MBCs comprised a variable proportion of the total spike-specific MBC response (4.6%–1.0%; median, 19.3%), the remainder representing B cells targeting non-RBD regions of the spike protein. The proportions of RBD- and non-RBD-binding, spike-specific MBCs again showed no changes with age (Figure 2I).

### Skewed isotype and activated memory phenotype of spike- and RBD-specific B cells.

**Figure 3:**
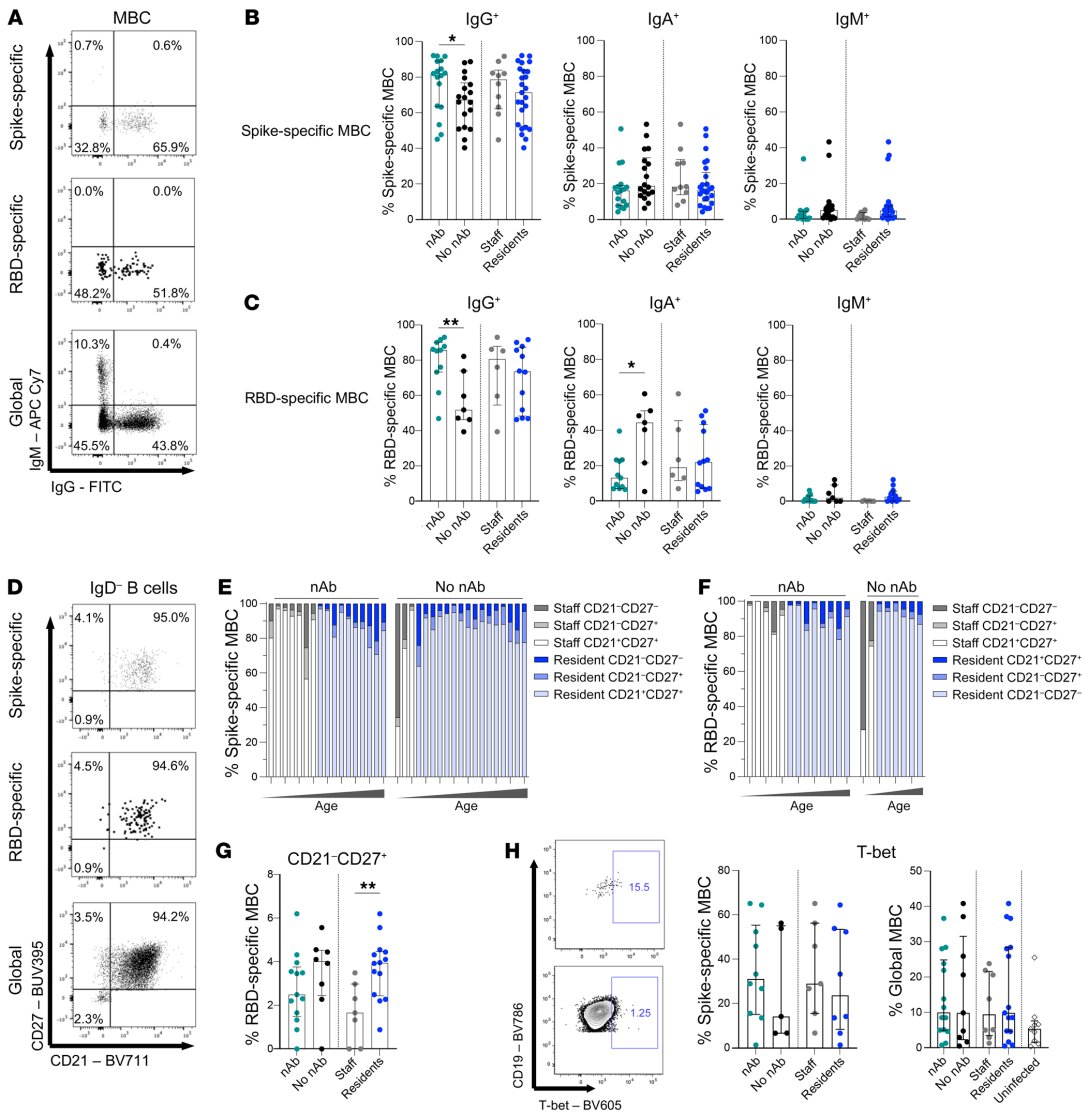
Preserved memory phenotype but skewed isotype of spike- and RBD-specific B cells with loss of nAbs. (**A**) Representative FACS plots of IgM/IgG on spike-specific, RBD-specific, and global MBCs from an infected individual. (**B**) Frequency of IgG^+^, IgA^+^ (IgD^–^IgG^–^IgM^–^), and IgM^+^ spike–specific MBCs by nAbs (*n =* 17) and no nAbs (*n =* 18) at 5 months and staff (gray, *n =* 10) and resident (blue, *n =* 25) status. (**C**) Frequency of IgG^+^, IgA^+^, and IgM^+^ RBD–specific MBCs by nAbs (*n =* 11) and no nAbs (*n =* 7) at 5 months and by staff (gray, *n =* 6) and resident (blue, *n =* 12) status. (**D**) Representative FACS plots of CD21 and CD27 on spike-specific, RBD-specific, and global MBCs from an infected individual. (**E**) Frequency of CD21^–^CD27^+^, CD21^+^CD27^+^, and CD21^–^CD27^–^ subsets of spike-specific MBCs by nAbs (*n =* 17) and no nAbs (*n =* 19) at 5 months, ordered by increasing age. (**F**) Frequency of CD21^–^CD27^+^, CD21^+^CD27^+^, and CD21^–^CD27^–^ subsets of RBD-specific MBCs with nAbs (*n =* 13) or no nAbs (*n =* 8) at 5 months, ordered by increasing age. (**G**) Frequency of CD21^–^CD27^+^ RBD-specific MBCs by nAbs (*n =* 13) and no nAbs (*n =* 8) at 5 months and by staff (gray, *n =* 7) and resident (blue, *n =* 14) status. (**H**) Representative plots and summary data showing the frequency of spike-specific and global MBCs expressing T-bet, by nAbs (*n =* 19) and no nAbs (*n =* 13) at 5 months, by staff (gray, *n =* 10) and resident (blue, *n =* 22) status, and by uninfected controls (*n =* 13). (**B**,** C**, and **G**) Bars indicate the median and IQR. A Mann-Whitney *U* test was used to determine statistical significance (*P < 0.05, **P < 0.005). (**B**) IgG *P =* 0.0382; NS, IgA *P =* 0.0045; NS, IgM. (**C**) IgG *P =* 0.0220; NS, IgA *P =* 0.0055; NS, IgM. (**G**) NS, *P* = 0.0180. (**H**) Bars indicate the median and IQR. Statistical significance was assessed by Kruskal-Wallis test with Dunn’s correction between nAb, no nAb, and uninfected subgroups and staff, resident, and uninfected subgroups, respectively, on global cell populations (all NS). Analysis was performed on all individuals with 50 or more cells in the parent gate for all phenotypic analyses.

Having identified and quantified antigen-specific B cells with tetramer staining, we were able to apply high-dimensional multiparameter flow cytometry to phenotype these low-frequency cell populations without any in vitro manipulation. We investigated the Ig isotype, memory phenotype, homing markers, and transcription factor usage of spike- and RBD-specific B cells and global B cells (Figure 3).

The vast majority of SARS-CoV-2 MBCs expressed IgG, with a similar isotype distribution observed between spike- and RBD-specific MBCs (Figure 3, A–C). However, individuals with persistent nAbs had a higher frequency of IgG isotype–expressing spike- and RBD-specific MBCs than did their counterparts who had lost nAbs (Figure 3, B and C), indicating the establishment of a robust, class-switched memory response in these individuals. In contrast, those whose nAbs had waned below detectable limits had lost more IgG and had a relative preservation of IgA class–switched, RBD-specific MBCs (Figure 3, B and C). IgM-expressing cells represented a small proportion of spike- or RBD-specific MBCs 5 months after infection, and their frequencies were comparable between groups (Figure 3, B and C). In the elderly residents, we observed a similar trend toward less IgG on the spike-specific MBCs but, overall, no significant skewing of Ig class–switching compared with younger staff (Figure 3, B and C). Global B cells showed the same pattern of expression of different Ig isotypes on their surface in SARS-CoV-2–resolved donors as in uninfected controls, with roughly equal proportions of IgG and IgA and less than 15% IgM (Supplemental Figure 3A).

We examined MBC subsets using the combination of CD27 and CD21. The majority of spike- and RBD-specific B cells had a classical resting memory phenotype (CD27^+^CD21^+^), characteristic of functional responses and comparable to the global MBC compartment, in both the elderly resident and staff groups (Figure 3, D–F, and Supplemental Figure 3B). Double-negative (DN) B cells have been associated with B cell dysfunction in aging (33–35) and the DN2 subset with an extrafollicular short-lived plasmablast response in the acute phase of a cohort with severe COVID-19 (36). However, at the 5-month time point following mild or asymptomatic infection in our cohort, neither the elderly nor those who had lost nAbs showed any expansion of CD27^–^CD21^–^ B cells (Figure 3, E and F) or the DN2 subset (CD27^–^CD21^–^CXCR5^lo^CD11c^hi^, Supplemental Figure 3C). Instead, we found a selective enrichment of the activated MBC subset (CD27^+^CD21^–^, previously described to be expanded in HIV and Ebola infection or after vaccination; refs. 37–39) in the RBD-binding fraction in elderly residents, with the same trend observed in those who had lost nAbs (Figure 3G). Those who had lost nAbs also had reduced expression of the B cell homing molecules CXCR3 and CXCR5 on spike-specific and global MBCs (nonsignificant trend and significant, respectively, Supplemental Figure 3, D–F). T-bet, a transcription factor critical for acute antiviral function in B cells but associated with dysfunction in chronic infections and autoimmunity (40–43), also tended to be expressed at lower levels in the spike-specific MBCs of those losing nAbs (Figure 3H).

Taken together, the isotype and memory phenotype of global and antigen-specific B cells was largely preserved in the elderly care home population, apart from a notable increase in spike-specific activated MBCs. Individuals who maintained nAbs had predominantly IgG-expressing antigen-specific MBCs. In contrast, in those who had lost nAbs by 5 months, whether staff or residents, residual antigen-specific B cells showed preferential preservation of IgA.

### Elderly residents maintain functional spike- and RBD-specific B cells but at reduced frequency compared with younger care home staff members.

Having found that antigen-specific MBCs could persist following loss of all detectable circulating nAbs, we wanted to confirm their potential for functional recall upon reencountering SARS-CoV-2. We therefore used cultured B cell enzyme-linked immune absorbent spots (ELISPOTs) to examine the in vitro capacity of persistent SARS-CoV-2–specific MBCs to differentiate into plasmablasts capable of secreting IgG-binding recombinant trimeric spike, S1 subunit of the spike protein (S1), or RBD proteins.

ELISPOTs were performed using PBMCs from 32 seropositive elderly care home residents and staff members (*n* = 23 residents, *n* = 9 staff), with the threshold for detection set at the highest observed value in an uninfected control group (*n* = 5 seronegative elderly care home residents and *n* = 5 pre-pandemic controls). Only individuals with responses detectable in a control total IgG well were included in the analysis. Where responses were too numerous to count (TNTC), we used the highest number of spot-forming cells (SFCs) observed in the maximal response to the respective protein (Supplemental Figure 4A).

**Figure 4:**
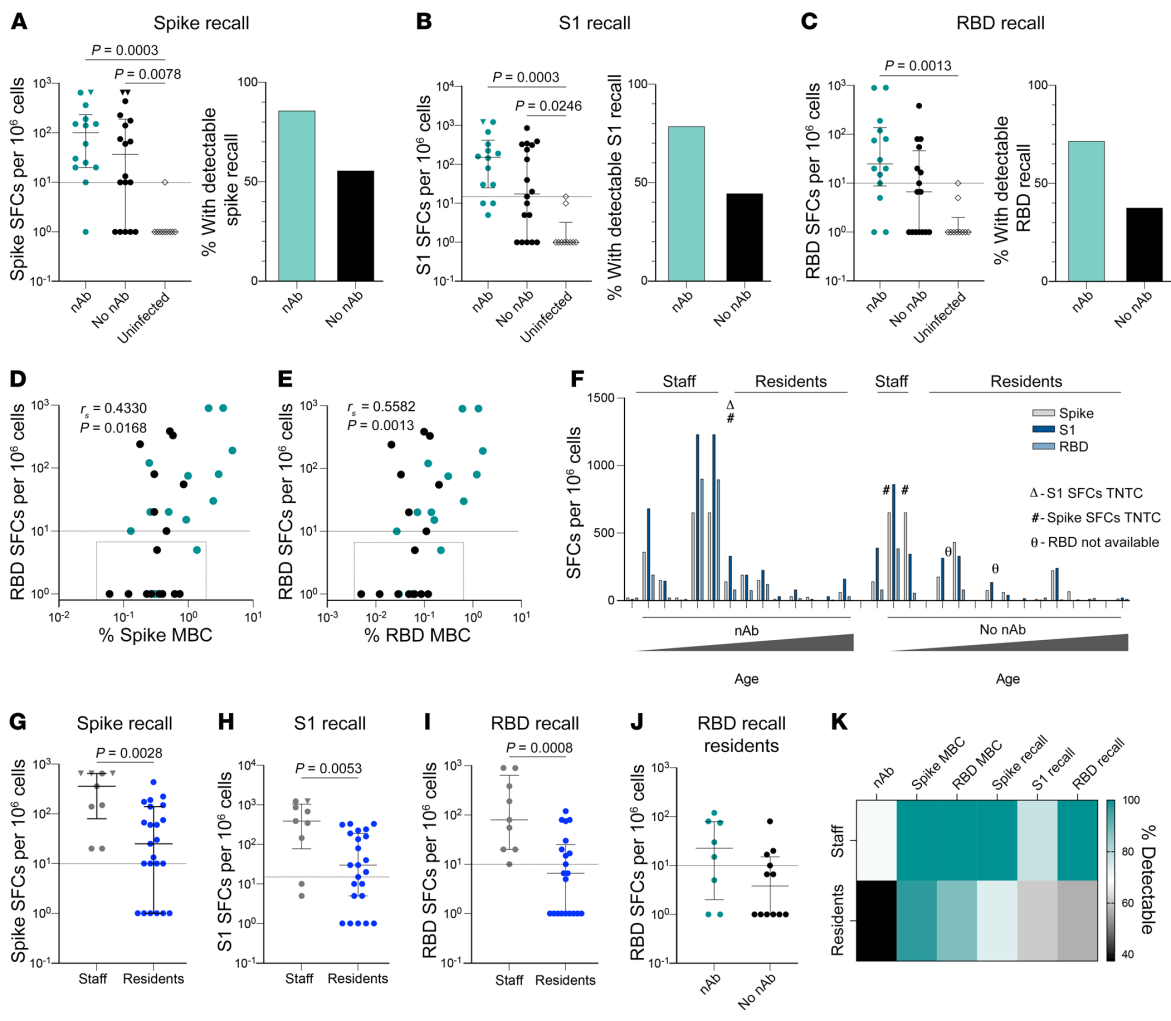
Elderly individuals maintain some functional spike- and RBD-specific B cells at reduced frequency compared with younger care home staff. (**A**–**C**) Left panels: SFCs/10^6^ PBMCs for infected individuals with nAbs or no nAbs at 5 months and for uninfected controls. Right panels: Proportion of infected individuals with detectable recall responses for (**A**) spike (nAbs, *n =* 14; no nAbs, *n =* 18; uninfected, *n =* 10), (**B**) S1 (nAbs, *n =* 14; no nAbs, *n =* 18; uninfected, *n =* 10), and (**C**) RBD protein (nAbs, *n =* 14; no nAbs, *n =* 16; uninfected, *n =* 10). (**D** and** E**) Correlation between RBD SFCs/10^6^ PBMCs and frequency of (**D**) spike-specific and (**E**) RBD-specific MBCs for those with (green) or without (black) nAbs. (**F**) SFCs/10^6^ PBMCs indicating recall responses for spike (gray), S1 (dark blue), and RBD (pale blue) per individual, stratified by nAb (*n =* 14) and no nAb (*n =* 18) status at 5 months and ordered by increasing age. (**G**–**I**) SFCs/10^6^ PBMCs for infected staff members and residents indicating recall responses for (**G**) spike (staff, *n =* 9; residents, *n =* 23), (**H**) S1 (staff, *n =* 9; residents, *n =* 23), and (**I**) RBD (staff, *n =* 9; residents, *n =* 21). (**J**) RBD SFCs/10^6^ PBMCs for infected residents with nAbs (*n =* 8) or no nAbs (*n =* 12) at 5 months. (**K**) Summary heatmap of the proportion of staff members and residents with nAbs, spike- and RBD-specific MBCs by flow cytometry, and spike, S1, and RBD recall by ELISPOT at 5 months. Bars in **A**–**C** (left panels) and **G**–**J **indicate the median and IQR; dashed lines indicate the threshold for seronegative and pre-pandemic controls. Statistical significance in **A**–**C** (left panels) was determined by Kruskal-Wallis test with Dunn’s correction. Statistical significance in **A**–**C** (right panels) was determined by Fisher’s exact test; (**A**) *P =* 0.3413, (**B**) *P =* 0.3926, and (**C**) *P =* 0.1870. (**D** and** E**) Dot-outlined boxes indicate individuals with a discordant MBC and ELISPOT response. Statistical significance was determined by Spearman’s rank correlation. (**G**–**J**) Statistical significance was evaluated by Mann-Whitney *U* test. (**A**, **B**, **G**, and** H**) Inverted triangles indicate TNTC responses, with the maximal response observed assigned. (**F**) Δ indicates S1 SFCs TNTC; # indicates spike SFCs TNTC; and θ indicates that RBD counts were unavailable. Individuals with zero response were assigned a value of 1 for logarithmic plotting; statistical analysis was performed using original values.

We observed functional recall responses to SARS-CoV-2 trimeric spike protein in 26 of the 32 seropositive individuals tested, with ELISPOTs tending to be positive in a larger number of those who had maintained nAbs (Figure 4A). However, the majority of individuals who had lost detectable nAbs still had a spike-specific response by ELISPOT, with no significant difference in their magnitude compared with the nAb group (Figure 4A). ELISPOTs revealed similar results for binding of IgG to S1 and RBD, with a trend toward a lower proportion of positive results in individuals who had lost nAbs, but no significant difference in the magnitude of B cell recall responses in those who did or did not maintain serum nAbs (Figure 4, B and C).

The magnitude of the RBD recall response assessed by ELISPOT showed a significant correlation with detection of both spike and RBD proteins in MBCs by tetramer staining (Figure 4, D and E). However, there was some discordance due to individuals who had tetramer-binding spike or RBD B cells that did not produce detectable IgG by ELISPOT (dot-outlined boxes, Figure 4, D and E), mainly in those who had lost nAbs. IgM- and IgA-specific ELISPOTs detected minimal numbers of S1 and RBD IgM- and IgA-expressing cells in the subset of individuals who had negative IgG ELISPOTs despite detectable tetramer binding (Supplemental Figure 4, B and C), suggesting that isotype specificity was not the main factor accounting for this discrepancy. Importantly, these data revealed that circulating antigen-specific B cells can be detected in the absence of functional recall.

Next, we compared functional responses to all 3 proteins for each individual, ranked according to nAb status and age. Individuals with strong recall responses to spike (as measured by ELISPOT) tended to also have strong responses to S1 and RBD, whereas others had weak responses to all 3 antigens (Figure 4F). Functional MBC recall responses decreased with increasing age in both the groups, regardless of whether serum nAbs were maintained (Figure 4F). Thus, elderly residents had significantly lower ELISPOT MBC responses against spike, S1, and RBD than did the younger staff group (Figure 4, G–I), which was confirmed with a negative correlation between age and ELISPOT response to all 3 antigens (Supplemental Figure 4, D–F). Focusing on elderly residents, we found that those who had lost nAbs tended to have undetectable or reduced MBCs capable of a functional recall response to RBD (Figure 4J).

Overall, the measurement of nAbs against live virus, combined with the assessment of spike- and RBD-specific MBCs by tetramer staining and functional ELISPOTs, provided complementary insights into B cell immunity (Figure 4K). A substantial proportion of those who had lost detectable neutralizing activity against live virus maintained spike- and RBD-specific MBCs detectable with 1 or both assays, regardless of age. However, some of those with persistent antigen-specific MBCs could not mount a detectable functional response, particularly the elderly (Figure 4K).

## Discussion

In this study, we sampled a cohort of very elderly residents and younger staff who developed mild or asymptomatic SARS-CoV-2 infection during care home outbreaks, a high proportion of whom had lost nAbs by 5 months (despite the maintenance of spike-binding Abs). This allowed us to dissect the potential for B cell memory to persist beyond detectable serum nAb levels, providing a backup reserve for humoral immunity. We demonstrated by flow cytometry that the majority of the cohort maintained detectable frequencies of spike- and RBD-specific MBCs, even when they had lost circulating Abs capable of live virus neutralization. Tetramer staining allowed accurate ex vivo quantification and characterization of antigen-specific MBCs, revealing that individuals who had lost detectable nAbs had lower frequencies of spike- and RBD-specific MBCs, with a preserved classical memory phenotype but class-switching skewed away from IgG toward IgA. Elderly and younger recovered individuals infected in the same care home outbreaks maintained similar frequencies of spike- and RBD-specific tetramer-staining B cells, with comparable isotypes but an increase in activated RBD-specific MBCs in the elderly. Importantly, functional assessment using ELISPOT assays demonstrated that the persisting spike, and particularly RBD-specific, MBCs had reduced potential for Ab production in the elderly.

The success of an infection or vaccine at inducing durable humoral immunity is dependent on the generation of long-lived plasma cells and MBCs (17–19). The longevity of the plasma cell response, capable of sustaining Abs, varies widely following different viral infections (5–7). A recent study demonstrated the presence of bone marrow plasma cells secreting IgG against SARS-CoV-2 spike protein in 15 of 19 individuals examined 7 months after infection (44), a finding in line with the durability of some Abs in the first year after mild infection. Nevertheless, many studies have also highlighted the potential for nAbs against SARS-CoV-2 to wane to a point where there is an, as yet ill-defined, risk of reinfection (45–47). Our study deliberately focused on the role of MBCs in individuals with waning or undetectable nAbs against live virus, despite the persistence of binding Abs. MBCs, previously identified in younger COVID-19 cohorts (11, 26, 48, 49), can provide crucial backup by responding quickly to a pathogen re-encounter or vaccination to form new plasmablasts, producing potent affinity-matured Abs with more flexible recognition of viral variants (9, 20); this is consistent with the enhanced nAb response described following vaccination of health care workers previously infected with SARS-CoV-2 (50). Our demonstration that B cells of relevant specificities can still be detected even when nAb titers are waning or completely undetectable provides some reassurance that a memory response remains intact in the elderly. Future large-scale studies are needed to assess whether B cell memory serves as an independent correlate of protection, or whether reliance on MBCs to mount a new response in the absence of existing Abs provides a critical window of opportunity for a virus that replicates as rapidly as SARS-CoV-2.

One strategy to combat Abs that are waning or unable to cross-recognize emerging variants is the use of booster vaccines. Our finding that the elderly have impaired differentiation of persistent spike- and RBD-specific MBCs into Ab-producing cells, as determined by ELISPOT assays, provides a biological rationale for the potential need for more frequent booster vaccinations in this high-risk group. The frequency and class-switching responses of antigen-specific B cells did not reveal obvious changes in the elderly group that would account for this functional defect, but phenotypic analysis of MBCs did reveal an increase in the CD27^+^CD21^–^ subset. The activated CD27^+^CD21^–^ subset of MBCs has recently been noted to remain expanded in some resolved COVID-19 patients (51), consistent with emerging literature supporting the possibility of prolonged antigen persistence, exemplified by a recent study detecting SARS-CoV-2 in the small bowel 4 months after asymptomatic infection (9). Our finding of an expanded population of CD27^+^CD21^–^ MBCs selectively within RBD-specific (and not global) responses in the older age group raises the possibility there is more prolonged antigen persistence and resultant B cell activation following SARS-CoV-2 infection in the elderly. The aging immune system is characterized by a tendency toward low-level chronic inflammation (52, 53), which could also contribute to prolonged activation; however, this might be expected to affect all MBCs irrespective of antigen specificity. Analogous to our findings in elderly care home residents, both older individuals and those with HIV have been found to have persistent circulating MBCs but defective plasmablast formation, resulting in reduced influenza vaccine–induced Abs (54, 55). Such age-related defects in B cell responses to vaccination have been attributed to a combination of B cell–intrinsic senescence and defective Tfh cells in germinal centers (56–58).

One limitation of our study is that the majority of care home residents and staff were female, implying that results cannot necessarily be generalized to males. Residents and staff were matched by the fact they were infected during the same care home outbreaks, therefore likely with the same SARS-CoV-2 strain and time frame. A potential confounder may be that more residents than staff members were symptomatic, although all symptomatic individuals in both groups were mild, with no requirement for oxygen or hospital attendance. Data on other cofactors that could have influenced immunity in addition to age were not available; larger cohorts would be needed to assess these. Another caveat to our study is that we were only able to study circulating B cells, whereas additional recall responses may be compartmentalized within the mucosa. A recent study suggested that mild infection can stimulate mucosal SARS-CoV-2–specific IgA secretion in the absence of circulating Abs (59). The bias toward the retention of IgA^+^ spike- and RBD-specific MBCs in those who had lost all detectable serum nAbs against live virus could therefore be reflective of a stronger mucosal response in these individuals. An increase in mucosal-homing IgA responses has been described as a feature of the aging immune response (60), consistent with the older composition of our cohort. Alternatively, the relative preservation of IgA rather than IgG spike- and RBD-specific MBCs in those with the fastest waning nAbs may simply reflect the recent observations that IgA dominates the early nAb response to SARS-CoV-2 infection and may not decline as fast as the IgG response (9, 61). In vitro ELISPOT assays may underestimate the full extent of residual SARS-CoV-2–specific responses able to mount functional memory in vivo. In addition, several studies have shown that the magnitude of the MBC response to SARS-CoV-2 continues to increase beyond 6 months (9, 23, 51, 62), again implying that we may have underestimated the extent of recall potential in our cohort at 5 months. Future studies should also examine the preservation of non-spike-specific MBCs with the potential to produce Abs mediating antiviral effects beyond neutralization, since other viral proteins (ORF3a, membrane and nucleocapsid) can play a dominant role in triggering Ab-dependent NK cell activation (63).

In conclusion, by focusing on an elderly cohort with a high proportion of nAb loss, we demonstrated that this waning in the first line of humoral defense could be compensated by the presence of a reserve of adaptive B cell memory in the majority of cases. Our findings highlight the importance of including measures of B cell memory in larger studies of natural infection and vaccination to determine their role as additional correlates of protection. Our data underscore the idea that identifying antigen-specific B cells by tetramer antigen staining is useful for quantitation and thorough ex vivo characterization, but may not necessarily equate with the preservation of a functional response, in line with discrepancies between the frequency and function of MBCs described in chronic viral infection (43, 64). The relative preservation of IgA antigen–specific MBCs in those with waned serum nAb raises the possibility that mucosal sequestered immunity may outlast that which is detectable in the circulation. Increased expansion of activated MBCs in the elderly highlights the need to investigate whether these cells are more prone to prolonged stimulation from persistent reservoirs of SARS-CoV-2 antigen. A finding of concern was the lack of detectable functional recall to RBD in elderly donors who had lost nAbs; given that RBD is the dominant site for nAbs, this observation supports the need for additional monitoring and/or booster vaccines to maintain sufficient Abs to neutralize emerging variants in this highly vulnerable group.

## Methods

### Participants.

SARS-CoV-2 antigen–specific MBC responses were compared between elderly care home residents and younger staff counterparts exposed to the virus in the same environment. Individuals from 6 care homes that reported SARS-CoV-2 outbreaks to Public Health England (PHE) were recruited for longitudinal SARS-CoV-2 reverse transcription PCR (RT-PCR) and serological follow-up in April 2020 (T0; refs. 1, 21). The serostatus of the individuals in these care homes at 1 month and 5 months after the outbreaks was established using binding and functional assays as previously described (21, 22). Briefly, seropositivity was assessed with a native virus lysate ELISA assay, RBD binding, and virus neutralization using the England 2 SARS CoV-2 prototype virus (21, 22).

A total of 42 individuals with mild or asymptomatic SARS-CoV-2 infection (*n* = 32 elderly residents; *n* = 10 staff members), all of whom were seropositive according to at least 1 of the binding assays described above at both sampling time points (month 1: May 2020, month 5: September 2020; Supplemental Table 2), were recruited along with 11 control SARS-CoV-2–seronegative individuals from 3 of the care homes. Participants donated 30 mL blood to be processed for PBMCs and serum 5 months after the initial outbreaks (month 5). Stored pre-pandemic samples from 7 healthy individuals were used as controls.

### Sample processing and data collection.

Venepuncture blood samples collected in lithium heparin–coated tubes, and serum separation tubes were used for isolation of PBMCs and serum, respectively. PBMCs were isolated by density centrifugation using Pancoll human (PAN-Biotech). Isolated PBMCs were frozen in FBS supplemented with 10% DMSO (MilliporeSigma). Prior to use, samples were thawed and washed in PBS. Serum was collected following centrifugation and stored at –80°C prior to use.

Clinical and laboratory data including age, sex, symptoms, and SARS-CoV-2 RT-PCR status at the time of the initial outbreak were available for participants (Supplemental Table 1 and ref. 1).

### Protein expression and purification.

Recombinant spike and spike RBD proteins of SARS-CoV-2 for antigen-specific B cell flow cytometry and ELISPOT were expressed and purified as previously described (15). Briefly, spike glycoprotein trimer (uncleaved spike stabilized in the prefusion conformation (GGGG substitution at furin cleavage site and 2P mutation; ref. 65) and RBD protein (12) were cloned into a pHLsec vector containing Avi and 6xHis tags. Biotinylated spike and RBD were expressed in Expi293F cells (Thermo Fisher Scientific). Supernatants were harvested after 7 days and purified. For the production of biotinylated protein, spike- and RBD-encoding plasmids were cotransfected with BirA and PEI-Max in the presence of 200 μM biotin.

Recombinant S1 protein constructs spanning SARS-CoV-2 residues 1–530 for ELISPOT were produced as previously described (28, 30). Briefly, codon-optimized DNA fragments were cloned into mammalian expression vector pQ-3C-2xStrep to create plasmids, which were then transfected into Expi293F cells growing at 37°C in a 5% CO_2_ atmosphere using ExpiFectamine reagent (Thermo Fisher Scientific). Proteins were purified by strep-tag affinity followed by size exclusion chromatography.

### Flow cytometry.

High-dimensional, multiparameter flow cytometry was performed for ex vivo identification of spike- and RBD-specific B cells. Two panels (surface and intranuclear) of mAbs were used to phenotype global and antigen-specific subsets (Supplemental Table 3). Biotinylated tetrameric spike (1 μg) and RBD (0.5 μg) were fluorochrome linked for flow cytometry by incubation with streptavidin-conjugated allophycocyanin (APC) (ProZyme) and phycoerythrin (PE) (ProZyme) for spike and with BV421 (BioLegend) for RBD, for 30 minutes in the dark on ice.

PBMCs were thawed and incubated with Live/Dead fixable dead cell stain (UV, Thermo Fisher Scientific) and saturating concentrations of phenotyping mAbs (Supplemental Table 2) diluted in 50% 1× PBS 50% Brilliant Violet Buffer (BD Biosciences). For identification of SARS-CoV-2 antigen–specific B cells, 1 μg per 500 μL stain each of tetrameric spike-APC and spike-PE and 0.5 μg per 500 μL stain of tetrameric RBD-BV421 were added to the cell preparation. Parallel samples stained with an identical panel of mAbs, but excluding the SARS-CoV-2 proteins (fluorescence minus one [FMO] controls), were used as controls for nonspecific binding.

Cells were incubated in the staining solution for 30 minutes at room temperature, washed with PBS, and subsequently fixed with either fixation and permeabilization solution (BD Biosciences) or a FOXP3 Buffer Set (BD Biosciences) according to the manufacturer’s instructions for surface and intranuclear staining, respectively. Saturating concentrations of mAbs diluted in 1× PBS were added following permeabilization for the detection of intranuclear proteins. All samples were acquired on a Fortessa-X20 (BD Biosciences) and analyzed using FlowJo (TreeStar).

B cell subsets were defined as CD19^+^CD20^+ ^MBCs, excluding the IgD^+^, CD38^hi^, and CD21^+^CD27^–^ naive fractions (see gating strategy in Supplemental Figure 1A) and CD19^+^CD20^+^CD38^+/–^CD21^–^CD27^–^CD11c^hi^CXCR5^lo^ DN2 cells. For analysis of RBD-costaining cells, a sufficient magnitude of spike-specific MBCs (≥20 dual spike^+^ cells) was required. For phenotypic analysis of spike- and RBD-specific cells, a sufficient magnitude of responses (≥50 cells in the relevant parent gate) was required.

### MBC recall response to SARS-CoV-2 by ELISPOT.

To activate MBC differentiation, 1 × 10^6^ PBMCs were stimulated with 1 μg/mL R848 (TLR7/8 agonist; resiquimod, InvivoGen) diluted in complete RPMI (cRPMI) (RPMI supplemented with 10% FBS plus recombinant human IL-2; 20 IU/mL; Peprotech), as previously described (66, 67). Activated cells were incubated for 6 days with a media change on day 3.

ELISPOT plates (Mabtech) were precoated with recombinant SARS-CoV-2 trimeric spike (1 μg/mL), S1 (1 μg/mL), and RBD (10 μg/mL) and anti–human IgG (1 μg/mL, Jackson ImmunoResearch) overnight at 4°C. Coated plates were blocked with cRPMI with 10% FBS prior to the addition of cells. Cultured PBMCs were added at varying concentrations depending on SARS-CoV-2 antigen and incubated at 37°C in 5% CO_2_ for 18 hours: 50,000 cells/ well to detect spike-specific, IgG-secreting cells; 100,000 cells/well to detect S1 and RBD IgG-secreting cells; and 1000 cells/well to detect total IgG-secreting cells. To control for nonspecific binding, uncoated control wells were incubated with 100,000 prestimulated cells. The following day, ELISPOT plates were washed in filtered PBS supplemented with 0.5% Tween-20 (Merck) and incubated for 4 hours in the dark at room temperature with 1 μg/mL goat anti–human IgG HRB Ab (Jackson ImmunoResearch). Cells were again washed 3 times with PBS–Tween-20 (0.5%) and 3 times with PBS, and then developed with 3-amino-9-ethylcarbazole (AEC) substrate (BD Biosciences) according to the manufacturer’s instructions. ELISPOT plates were washed with ddH_2_0 before analysis using ViruSpot (Autoimmun Diagnostika). All conditions were performed in duplicate and the responses averaged.

For the detection of IgM- and IgA-secreting cells, PBMCs were stimulated as before for 5 days. Cultured PBMCs were added to coated and blocked plates, as described above, and incubated at 37°C in 5% CO_2_ for 6 hours. ELISPOT plates were then washed in filtered PBS supplemented with 0.5% Tween-20 (Merck) and incubated overnight in the dark at 4°C with 1 μg/mL goat anti–human IgM- or IgA-HRP Ab (Jackson ImmunoResearch). Procedures under all conditions were performed in duplicate and the responses averaged. A control well coated with anti-IgM or anti-IgA was used for the detection of total IgM- or IgA-secreting cells. Data are presented minus the background, calculated from an uncoated well.

### Statistics.

Data were analyzed using GraphPad Prism (GraphPad Software). Descriptive statistical analyses were performed. Continuous data that did not follow a normal distribution were described as medians with IQRs, and differences were compared using the Mann-Whitney *U* test (2-tailed), Wilcoxon’s paired *t* test (2-tailed), or the Kruskal-Wallis test with Dunn’s post hoc test for pairwise multiple comparisons as appropriate. Contingency table analyses were conducted using Fisher’s exact test. Correlations for nonparametric data were assessed using Spearman’s rank correlation with a 95% CI. A *P* value of less than 0.05 was considered significant.

### Study approval.

The study protocol was reviewed and approved by the PHE Research Ethics and Governance Group (REGG reference NR0204). Written information regarding the study was provided to all participants. Verbal informed consent for testing was obtained by care home managers from staff members and residents or their next of kin as appropriate. Stored pre-pandemic samples from 7 healthy individuals were used as controls, recruited under ethics number 11/LO/0421 and approved by the South East Coast – Brighton and Sussex Research Ethics Committee.

## Author contributions

AJS, MZ, LEM, and MKM conceptualized the study. AJS, ARB, LM, KJD, SNL, LEM, and MKM designed the methodology. AJS, ARB, SL, JD, MP, RG, CRS, and LEM performed experiments. AJS, FA, SNL, and JYC collected samples and clinical data. AJS, ARB, SL, MP, RG, LEM, and MKM analyzed the data. Funding acquisition: SNL, JYC, MZ, and MKM acquired funding. SNL, MZ, LEM, and MKM supervised the study. AJS and MKM wrote the original draft of the manuscript. All authors reviewed and edited the manuscript.

## Supplementary Material

Supplemental data

## Version 1 11/29/2021

In-Press Preview

## Version 2 01/18/2022

Electronic publication
