# Supplementary material for: SARS-CoV-2–specific memory B cells can persist in the elderly who have lost detectable neutralizing antibodies
Source: J Clin Invest. 2022 Jan 18;132(2):e152042. doi: 10.1172/JCI152042 (PMC8759779; doi:10.1172/JCI152042)
Supplement: Supplemental data [file jci-132-152042-s126.pdf]

Supplementary Data

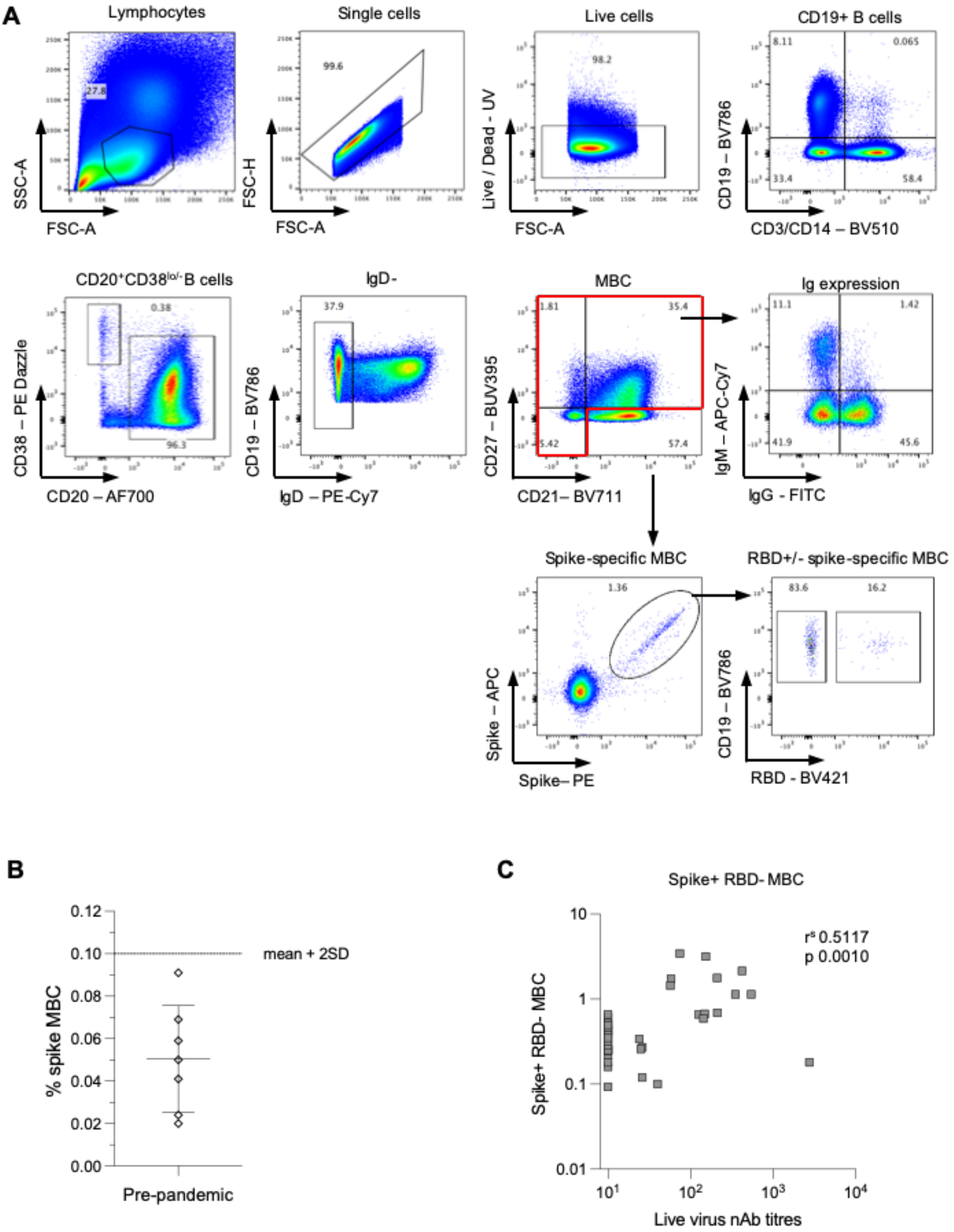

Figure S1b. Gating strategy and threshold for detection of spike-specific responses

(**A**) Representative FACS plot showing gating strategy to determine populations indicated. Spike- and RBD-specific gating applied to IgD- MBC gate. (**B**) Frequency of spike-specific MBC in pre-pandemic samples used to determine threshold for detectable levels of spike-specific by calculating mean + 2SD (n=7). (**C**) Correlation of frequency of spike-specific RBD- negative MBC and live virus nAb for all individuals with ≥20 cells in spike-specific gate (n=29). (**B**) Bars indicate mean and standard deviation. (**C**) Spearman's rank correlation. MBC – Memory B cell; Ig – immunoglobulin; RBD – receptor binding domain; nAb – neutralising antibody

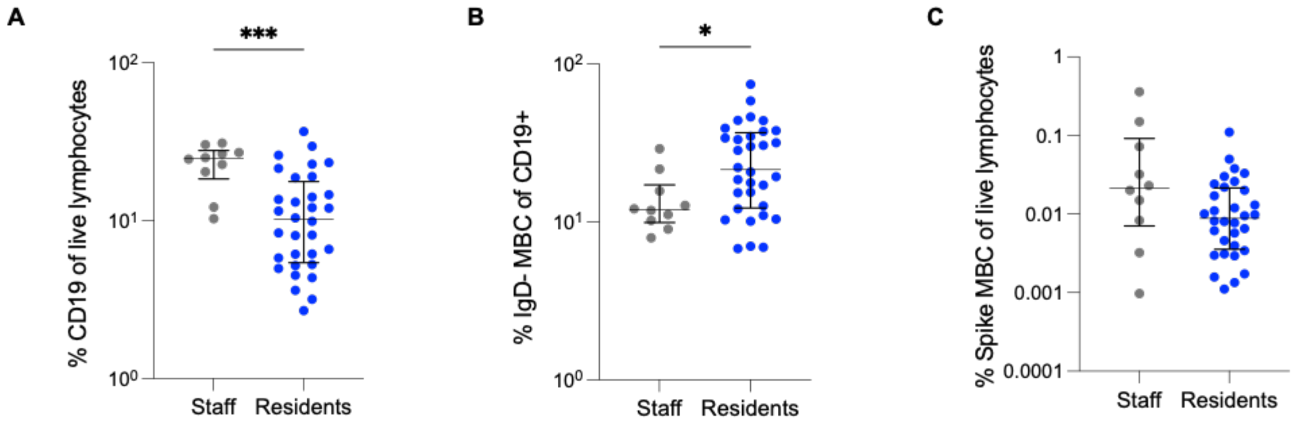

Figure S2. (**A**) Frequency of live, singlet CD19+ B cells in staff (n=10) and residents (n=32). Statistical analysis Mann-Whitney U test p=0.0008. (**B**) IgD- MBC (excluding CD21+CD27-) as a frequency of CD19+ B cells. Statistical analysis Mann-Whitney U test p=0.0265. (**C**) Spike specific MBC as a frequency of live lymphocytes. Statistical analysis Mann-Whitney U test p=0.0967.

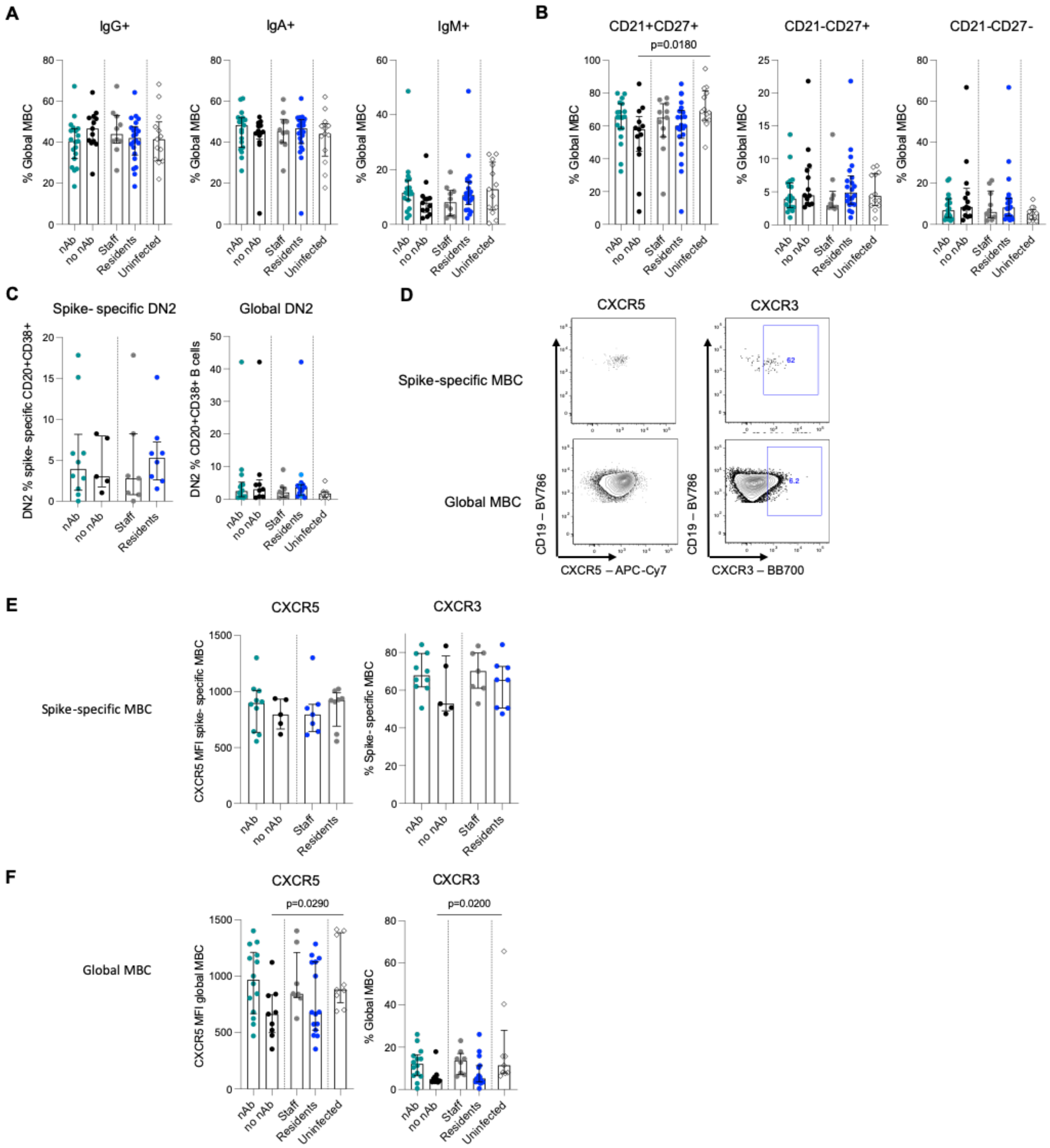

Figure S3. Phenotyping of spike-specific and global MBC

(**A**) Summary data of expression of immunoglobulin isotypes IgG, IgA and IgM on global MBC stratified by presence (nAb, n=19) and absence (no nAb, n=13) of detectable nAb at Month 5, and by staff (grey, n=10) and resident (blue, n=22) status, and uninfected controls (n=13). (**B**) Summary data of frequency CD21-CD27+, CD21+CD27+ and CD21-CD27- global MBC subsets stratified by presence (nAb, n=19) and absence (no nAb, n=13) of detectable nAb at Month 5, and by staff (grey, n=10) and resident (blue, n=22) status, and uninfected controls (n=13). (**C**) Left panel: Frequency of spike- specific cells with a DN2 phenotype (CXCR5^{lo} CD11c^{hi} CD21-CD27-) as a proportion of CD20+CD38+ B cells stratified by presence (nAb, n=10) and absence (no nAb, n=5) of detectable nAb at Month 5, and by staff (grey, n=7) and resident (blue, n=8) status. Right panel: Frequency of global CD20+CD38+ B cells with a DN2 phenotype stratified by presence (nAb, n=19) and absence (no nAb, n=13) of detectable nAb at Month 5, and by staff (grey, n=10) and resident (blue, n=22) status, and uninfected controls (n=13). (**D**) Representative FACS plots of CXCR5 and CXCR3 on spike- specific MBC (top panel) and global MBC (bottom panel). (**E**) Summary data of expression of CXCR5 (MFI) and CXCR3 (frequency) of spike-specific MBC stratified by presence (nAb, n=10) and absence (no nAb, n=5) of detectable nAb at Month 5, and by staff (grey, n=7) and resident (blue, n=8) status. (**F**) Summary data of expression of CXCR5 (MFI) and CXCR3 (frequency) of global MBC stratified by presence (nAb, n=19) and absence (no nAb, n=13) of detectable nAb at Month 5, and by staff (grey, n=10) and resident (blue, n=22) status, and uninfected controls (n=13). (**A, B, C, F**) Bars indicate median and interquartile range; Kruskal Wallis test with Dunn's post hoc analysis for multiple comparisons between nAb, no nAb and uninfected subgroups and staff, resident and uninfected subgroups respectively on global populations; significance where indicated. (**C, E**) Bars indicate median and interquartile range; Mann Whitney U test comparison between nAb and no nAb and staff and resident subgroups respectively for spike-specific subsets; significance where indicated. Analysis of individuals ≥50 cells in the relevant parent gate for all phenotypic analysis.

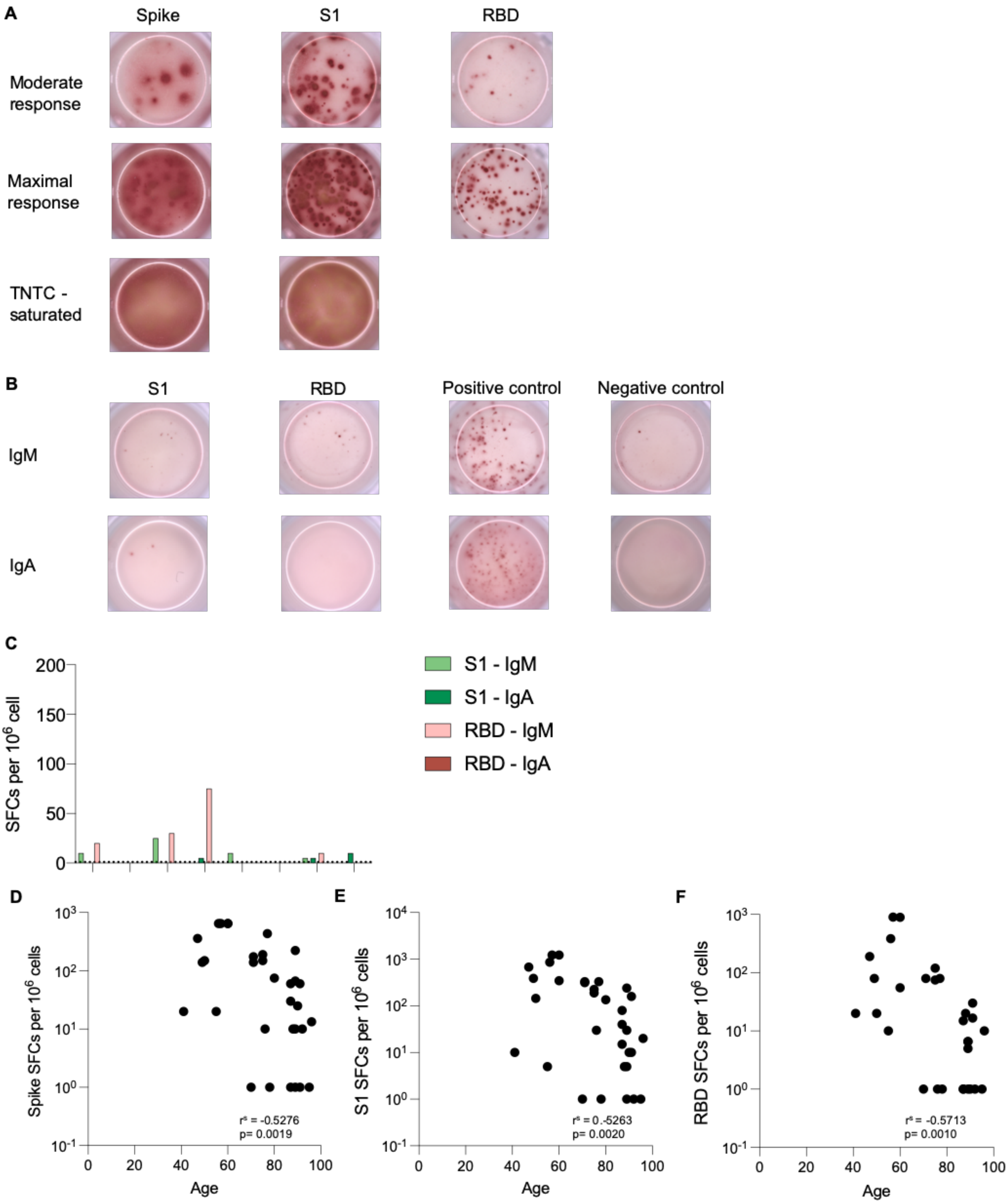

Figure S4. ELISpot responses to SARS-CoV-2 proteins

(**A**) Representative IgG ELISpot responses to SARS-CoV-2 Spike, S1, and RBD proteins. Top row demonstrates moderate response observed, middle row maximal responses, and bottom row saturated responses that were too numerous to count (TNTC). In the case of samples with saturated responses these individuals were assigned the value of the maximal response observed and highlighted as TNTC. (**B**) Representative images showing IgM- and IgA- responding SFCs targeting S1 and RBD proteins in residents with absent RBD-IgG recall responses. Positive (Total IgM/A) and negative (uncoated) wells are shown for comparison. (**C**) Enumeration of IgM- and IgA-producing SFCs per 1 million PBMC in residents with undetectable RBD-specific IgG+ recall responses (n=9), targeting RBD and S1 proteins. All frequencies are presented with background calculated from negative control wells subtracted. (**D-F**) Correlation between Age and Spike (n=32) (**D**), S1 (n=32) (**E**), and RBD (n=30) (**F**) SFCs per 10^6 PBMC. Spearman's rank correlation. Individuals with a zero response to any antigen have been assigned a value of 1 to allow plots to be drawn on a logarithmic scale. All statistical analysis performed using original values. SFC, spot forming cells; RBD, Receptor binding domain; MBC, memory B cells, TNTC, too numerous to count.

Table S1.

	Residents	Staff	Uninfected
Number	32	10	11
Age years (median; IQR)	87; 77-90	56; 50-60	82; 77-87
Sex (n, (%) female)	23 (71.9)	7 (70.0)	6 (54.5)
Symptomatic T0*	17	3	2
PCR positive T0	19/32**	5/9**	0/11
SARS-CoV-2 Seropositive T1***	32	10	0
SARS-CoV-2 Seropositive T2***	32	10	0

Cohort Characteristics

* Symptom status assessed during the 14 days before and at the initial outbreak was collected for all staff, who self-reported any symptoms, and residents, whose symptoms were recorded the care home staff. Daily interviews were undertaken with individual care homes to identify any newly symptomatic individuals for the subsequent 14 days. Typical COVID-19 symptoms at that time included fever 37.8^{o}C, shortness of breath or cough, while atypical symptoms included, but were not restricted to, new confusion, reduced alertness, fatigue, lethargy, reduced mobility and diarrhoea.

** Two individuals (1 staff and 1 resident) did not have a nasal swab for SARS-CoV-2 RT-PCR taken during the initial outbreak.

*** As assessed by native virus lysate assay (PHE) and / or receptor binding domain assay (RBD).

Table S2.

Month 1 Lysate	Month 5 Lysate	Month 1 RBD	Month 5 RBD	Month 1 nAb	Month 5 nAb
POS	POS	POS	POS	POS	POS
POS	NEG	POS	POS	POS	NEG
POS	POS	POS	POS	POS	POS
POS	POS	POS	POS	POS	NEG
POS	POS	POS	POS	POS	POS
POS	POS	POS	POS	NEG	NEG
POS	POS	POS	NEG	POS	NEG
POS	POS	POS	POS	POS	NEG
POS	POS	POS	POS	POS	NEG
POS	POS	POS	POS	POS	POS
POS	POS	POS	POS	POS	POS
POS	POS	POS	POS	POS	NEG
POS	POS	POS	POS	POS	NEG
POS	POS	POS	POS	POS	POS
POS	POS	POS	POS	POS	POS
POS	POS	POS	POS	POS	POS
POS	POS	POS	POS	POS	POS
POS	POS	POS	POS	POS	POS
POS	POS	POS	POS	POS	NEG
POS	POS	POS	POS	POS	POS
POS	POS	POS	NEG	POS	NEG
POS	POS	POS	POS	POS	POS
POS	POS	POS	POS	POS	POS
POS	POS	POS	POS	POS	POS
POS	POS	POS	POS	POS	POS
POS	POS	POS	EQUIV	POS	NEG
POS	POS	POS	POS	POS	POS
POS	POS	POS	POS	POS	NEG
POS	POS	POS	POS	POS	NEG
POS	POS	POS	POS	POS	POS
POS	POS	POS	POS	POS	POS
POS	POS	POS	POS	POS	POS
POS	POS	POS	POS	POS	NEG
POS	POS	POS	EQUIV	POS	NEG
POS	POS	POS	EQUIV	POS	NEG
POS	POS	POS	POS	POS	NEG
POS	POS	POS	POS	POS	NEG
POS	POS	POS	POS	POS	NEG
POS	POS	POS	POS	POS	NEG
POS	EQUIV	POS	POS	POS	NEG
POS	POS	POS	POS	POS	NEG
POS	POS	POS	POS	POS	NEG

Cohort serology results

Binding (native viral lysate and RBD assays, PHE) at Month 1 and Month 5, and live virus neutralising antibody results at Month 1 and Month 5 for all individuals (n=42). POS: above assay threshold; EQUIV: assay equivocal range; NEG: below threshold.

Table S3.

Marker	Fluorochrome	Clone	Source	Cat. number
CD3	BV510	OKT3	Biolegend	317332
CD11c	FITC	B-ly6	BD	561355
CD13	BV510	M5E2	Biolegend	301842
CD19	BV786	HIB19	BD	740968
CD20	AF700	2H7	BD	560631
CD21	BV711	B-ly4	BD	563163
CD24	PE-CY7	ML5	Biolegend	311120
CD27	BUV395	L128	BD	563815
CD38	PE-CF594	HIT2	BD	562288
CD183 (CXCR3)	BB700	1C6/CXCR3	BD	566532
CD185 (CXCR5)	APC-Cy7	J252D4	Biolegend	356926
IgD	PE-Cy7	IA6-2	BD	561314
IgG	FITC	G18-145	BD	560952
IgM	APC-Cy7	MHM-88	Biolegend	314520
STREP	APC	-	Prozyme	PJ25S
STREP	PE	-	Prozyme	PJRS25
STREP	BV421	-	Biolegend	405226
T-bet	BV605	4B10	Biolegend	644817
Viability	UV	-	Invitrogen / Thermofisher	L23105

Antibody list
